# Awareness, concern and willingness to adopt biosecure behaviours: public perceptions of invasive tree pests and pathogens in the UK

**DOI:** 10.1007/s10530-017-1467-4

**Published:** 2017-06-19

**Authors:** Julie Urquhart, Clive Potter, Julie Barnett, John Fellenor, John Mumford, Christopher P. Quine, Helen Bayliss

**Affiliations:** 1 Centre for Environmental Policy, Faculty of Natural Sciences, Imperial College London, South Kensington Campus, London, SW7 1NA UK; 2 Department of Psychology, University of Bath, 10West, Bath, BA2 7AY UK; 3 Forest Research, Northern Research Station, Roslin, Midlothian EH25 9SY UK; 4 London, UK

**Keywords:** Invasive tree pests and diseases, Public perceptions, UK national survey, Biosecure behaviours, Public awareness

## Abstract

The growing incidence of invasive tree pest and disease outbreaks is recognised as an increasing threat to ecosystem services and human wellbeing. Linked to global trade, human movement and climate change, a number of outbreaks have attracted high public and media attention. However, there is surprisingly little evidence characterising the nature of public attentiveness to these events, nor how publics might respond to evolving outbreaks and the management actions taken. This paper presents findings from an online questionnaire involving 1334 respondents nationally-representative of the British public to assess awareness, concern and willingness to adopt biosecure behaviours. Despite revealing low levels of awareness and knowledge, the results indicate that the British public is concerned about the health of trees, forests and woodlands and is moderately willing to adopt biosecure behaviours. A key finding is that membership of environmental organisations and strong place identity are likely to engender higher awareness and levels of concern about tree pests and diseases. Further, those who visit woodlands regularly are likely to be more aware than non-visitors, and gardeners are more likely to be concerned than non-gardeners. Women, older respondents, those with strong place identity and dependence, members of environmental organisations, woodland visitors and gardeners were most likely to express a willingness to adopt biosecure behaviours. A comparison with findings from a survey conducted by the authors 3 years previously shows a decline over time in awareness, concern and willingness.

## Introduction and background

The growing incidence of new invasive tree pest and disease introductions into the UK and elsewhere has been linked to globalization, increased trade and transportation of live plants and wood products, human movement and climate change (Liebhold et al. 2012; Potter and Urquhart 2017). Evidence suggests such introductions are likely to have profound consequences for ecosystem services and human wellbeing (Boyd et al. 2013; Freer-Smith and Webber 2015). Some outbreaks have attracted intense public and media attention, such as Dutch elm disease in the UK in the 1970s (Tomlinson and Potter 2010), the Asian longhorn beetle (*Anoplophora glabripennis*) outbreak in New York (Haack et al. 1997) in the 1990s and the recent outbreak of Ash dieback (*Hymenoscyphus fraxineus*) in the UK (Heuch 2014). This attention often focuses on the potential impacts of the outbreaks, especially in terms of effects on biodiversity and landscape, and the effectiveness of the government in preventing new incursions or managing pests and diseases already established. As Sheremet et al. (2017) indicate, when public funds are used for disease and pest control programmes, it is important to consider public attitudes towards trees and woodlands and their preferences for mitigation efforts. However, currently there is little empirical evidence for policy makers to refer to in order to characterise the nature of public attentiveness to tree pest and disease outbreaks, nor how publics might respond to evolving outbreaks and management actions (Flint 2007). This is important when, for instance, anecdotal evidence around the Ash dieback outbreak in the UK suggests that policymakers and some stakeholders appeared to assume that there were high levels of public concern, perhaps on the basis of media coverage, when they made their case for government intervention in 2012.

Alongside considering how public opinion affects management and policy-making, such as a lack of support for chemical pest control or clear-felling as control measures (Sheremet et al. 2017), understanding how lay publics interpret and respond to risk events is important for risk communication. Raising awareness without triggering undue alarm (Timotijevic and Barnett 2006) may require tailoring notifications and information about risk to particular circumstances, interests and knowledge of a heterogeneous set of lay publics (Quine et al. 2011). This necessitates a greater understanding of publics and also the role of ‘trusted’ social groups in communication and the promotion of dialogue (Quine et al. 2011).

Two recent (2013 and 2014) national surveys have been conducted to assess UK public awareness and concern about invasive tree pests and diseases, as well as their willingness to adopt biosecure behaviours^1^ and accept management strategies (Bayliss and Potter 2013; Fuller et al. 2016). Both surveys found general levels of awareness of tree pests and diseases were low, but with high levels of concern about the impacts on tree and woodland health, along with a willingness to adopt biosecure behaviours and support for management actions against tree pests and diseases. Similar findings have been identified for awareness amongst stakeholder groups (Marzano et al. 2015) such as tree professionals (Marzano et al. 2016), landowners (Molnar et al. 2003), local residents (Flint 2007; McFarlane et al. 2006) and outdoor recreationists and tourists (Runberg 2011).

Less clear, however, is whether public perceptions about risks to tree health change over time. Public risk perceptions are dynamic and may shift in response to changes in the risk itself (or its management) (Flint 2007; Loewenstein and Mather 1990). In the case of tree health, we might hypothesise that awareness and concern were likely to be elevated in 2012–2013 following the intense media coverage surrounding Ash dieback at the time (Mccombs and Reynolds 2002). However, while the results of the 2013 survey suggest there was concern about tree health issues, levels of awareness were generally low, with similar findings nine months later in 2014. A deeper understanding of the changing nature of public perceptions of risk is therefore needed.

Judging the significance of a risk requires making sense of media coverage, official notifications and personal encounters. These perceptions are likely to be filtered through values and meanings attached to whatever is under threat and some argue that attachments to a place or locale are likely to be particularly influential (Masuda and Garvin 2006; Venables et al. 2012). Such emotional attachments (Tuan 1974) are often characterized as *place identity* and *place dependence.* Place identity is associated with experiences, memories and beliefs attributed to a place (Relph 1976), and place dependence relates to the suitability of a locale for particular needs or activities (Jorgensen and Stedman 2001). Place attachment may influence how risk is socially constructed and experienced in local environments, with cultural meanings related to places and landscapes and demographic factors mediating risk concerns. As Washer (2011, p. 510) argues in relation to perception of risks posed by infectious diseases, it is increasingly important to understand “how meaning-making goes on ‘on the ground’, rooted in the local culture and lived experience of the people whose lives are touched by these infections”.

Our aim in the work drawn on in this paper was to assess the degree to which the British public are aware of, and concerned about, tree pests and diseases, as well as their willingness to adopt biosecure behaviours. Specific objectives were to assess the influence of socio-demographic and lifestyle factors on attitudes, knowledge and willingness to adopt biosecure behaviours, including the role of place identity and place dependence in mediating risk concerns. We included a number of questions to investigate change over time through direct comparison with a survey in 2013 (Bayliss and Potter 2013).

## Methods

### Survey

An online questionnaire was conducted across a nationally-representative sample of the British public. The survey instrument was adapted from a survey in 2013 (Bayliss and Potter 2013) in order to allow us to compare responses at the time of survey with those previously obtained three years ago. Respondents were asked to make judgements about their level of knowledge of tree pests and diseases, their information and communication sources and their experience. Questions also captured their concerns about tree health risks and their willingness to adopt biosecure behaviours. Lifestyle/attitudinal questions asked about frequency of woodland and countryside visits, the importance of woodlands and trees, activities such as plant purchasing, membership of environmental organisations and personal attachments to place. Demographic variables included gender, age, location (region), level of education, employment category, income, living situation and ethnicity.

A combination of multiple choice and Likert-scale questions was used. The survey was deployed by a professional panel survey company (http://www.respondi.com) using an online survey tool. The target sample size was 1200 respondents over the age of 18 and nationally representative of the UK population. Respondent quotas were set in order to gain a representative sample in terms of gender, age group and region, according to the Office for National Statistics projections for 2015. Once quotas were met the questionnaire was closed to those groups. The survey was deployed over a week in April 2016 via Respondi’s panel of registered respondents. The final dataset consisted of 1348 completed surveys. Eight respondents were under 18 and so were removed from the dataset. In order to achieve a ‘public’ sample, a further six responses were removed as the respondents indicated a livelihood linked to forestry or horticulture, leaving a total of 1334 responses for analysis.

### Statistical analyses

Data were analysed using IBM Statistical Package for Social Sciences (version 22.0) software. Six analytical approaches were adopted: (1) basic descriptive statistics; (2) factor analysis of place attachment scales to establish *place identity* and *place dependence* variables used in further analysis^2^; (3) cross-tabulations using Chi square tests to investigate the relationship between variables; (4) factor analysis of the ‘concern’ variable to identify dimensions of concern (principal component extraction method was adopted with oblimin rotation with Kaiser normalization); (5) ordinal logistic regression modeling to identify the variables likely to influence awareness and concern; and (6) cross-tabulation using Chi square tests to determine significant differences between 2013 and 2016 survey datasets. As with our 2016 survey, the 2013 survey consisted of a nationally representative sample of 1000 individuals gathered via an online survey deployed by the panel survey company Toluna (https://uk.toluna.com) (Bayliss and Potter 2013). This stage of the analysis involved Chi square statistics to determine whether there were statistically significant differences between the datasets and, if so, what the nature of those differences might be. Where variables were comparable for statistical purposes, Chi square statistics were used. For other variables, percentages are cited as indicative of apparent (although not statistically verified) differences between the datasets.

## Results

### Sample profile

The age group categories and geographical distribution of respondents was largely representative of national figures, with 82.7% living in England, 4.8% in Wales, 8.8% in Scotland and 3.4% in Northern Ireland. 51.9% of respondents were women and 48.1% were men, close to the nationally representative figures of 48.8% and 51.2%. In terms of ethnic group, the majority of respondents (92.1%) were white, which is slightly higher than the national statistic of 87%. The highest proportion of respondents indicated they were retired (25.6%) or in junior managerial administrative or professional roles (19.9%). Respondents further indicated a range of income brackets and level of education (Table 4 in “Appendix”).

**Fig. 1:**
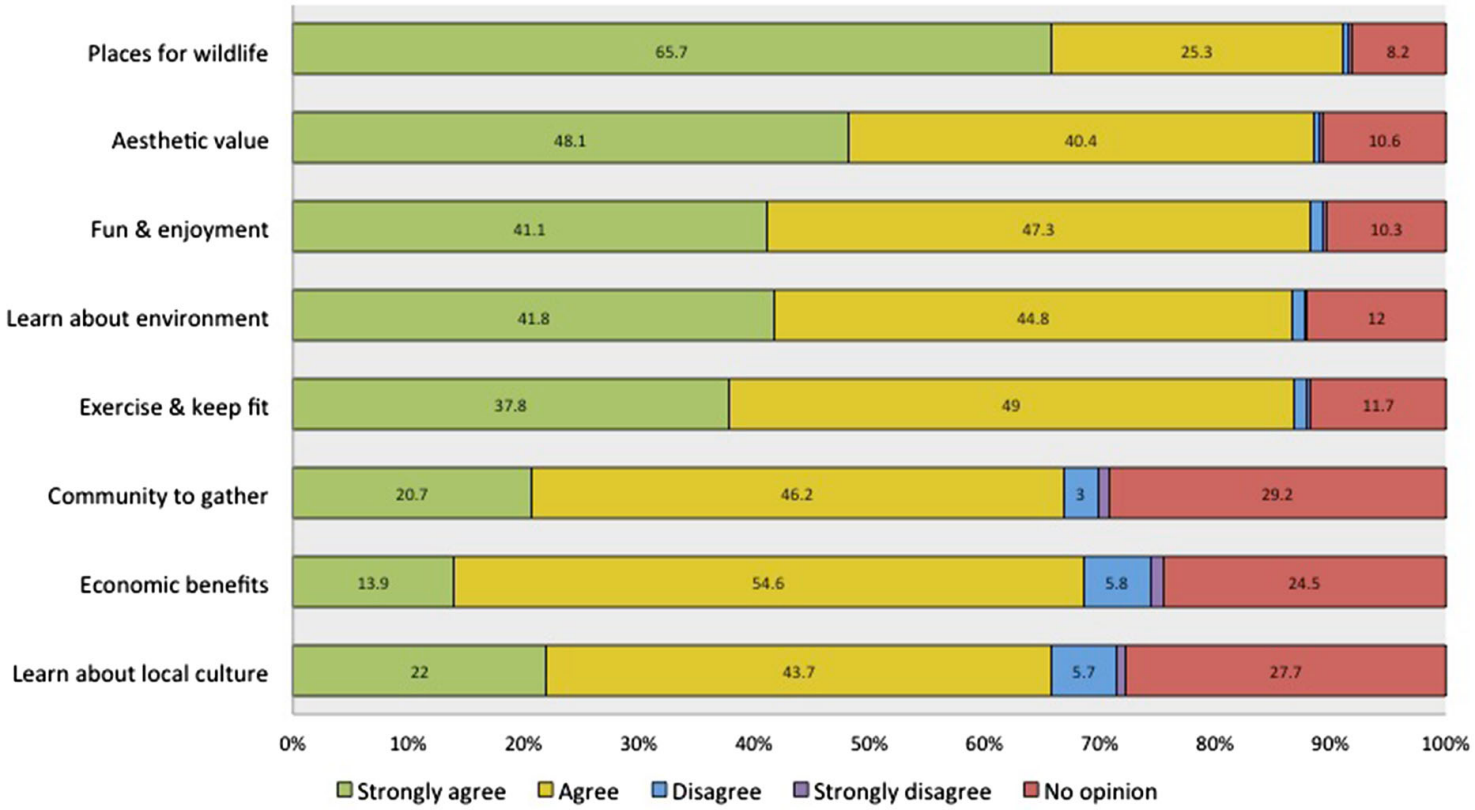
Importance of tree, woodlands and forests for a range of benefits

Respondents attached different levels of importance to a range of benefits provided by trees, woodlands and forests, with generally high agreement on all statements (Fig. 1). In addition, over half the respondents had visited a garden or park open to the public in the last 12 months (59.7%). 40.8% had visited more than one woodland or forest in the UK and only 13.8% had never visited woodlands or forests.

While 17.2% of respondents said they had exchanged plants with friends or family, only 4.9% had collected firewood from woodlands, and 1.0% had brought plants or untreated wood products home from a trip abroad. In terms of purchasing trees and plants, more indicated that they purchase plants than trees. The most popular place for buying trees was from a nursery or garden centre (8.0%) or supermarket (e.g. Homebase) (5.7%). Only 4.1% indicated that they buy trees from a local independent grower and 3.9% said they buy on the internet. Approximately a third (32.3%) of respondents said they had bought plants from a supermarket in the last 12 months. A further 26.9% had purchased plants from a nursery or garden centre, 15.3% from a local independent grower and 11.0% on the internet.

### Awareness and knowledge of tree health issues

Levels of awareness about tree pests and diseases were generally low, with 21.0% of respondents indicating that they had never heard of the issue, and a further 57.0% indicating they had heard of it but knew very little about the problem. Only 18.7% felt they are reasonably well informed about the issue, and 3.2% felt very well informed. Of those who had heard about pests and diseases, 72.8% said they had not been affected personally, while 20% said they had noticed an infected/infested tree in their neighbourhood, 10.4% indicated they have had to remove or treat a tree and 2.4% said they had volunteered as a citizen scientist.

Knowledge of specific tree pests and diseases varied greatly. Of those that had heard about tree pests and diseases, the most widely recognised of the pests and pathogens listed was Dutch elm disease (79.6%), followed by Ash dieback (44.3%), although around a third reported they had also heard of Acute oak decline (35.1%) and Asian longhorn beetle (32.3%). The least recognised was Massaria (4.8%), and *Phythophthora ramorum* was the second least known disease (5.4%). Other pests or diseases known about were Chestnut blight (28.4%), Large pine weevil (21.5%), Horse chestnut leaf miner (19.0%), Emerald ash borer (15.7%), Great spruce bark beetle (9.4%) and Dothistroma needle blight (7.1%).

The majority of those who reported they knew about tree pests and diseases said imported plants (85.4% very likely or likely), wood material (73.9%) and natural dispersal (73.9%) were the most likely pathways. Other pathways indicated included animals (73.7%), people (66.0%) and firewood or woodchips (53.9%).

There was a divergence of views about who has primary responsibility for managing and controlling tree pests and diseases, with 35.4% of respondents identifying the Forestry Commission (FC), 18.4% identifying the local authority and 10.6% saying the Woodland Trust (10.6%). A further 5.7% thought that woodland owners had responsibility and 21.4% did not know.

A majority (58.5%) of respondents indicated they did not have enough information to know what to do about tree pests and diseases. However, interest in learning more about the issue appears to increase significantly with current levels of awareness (*χ* ^2^(3) = 55.694, *p* < .001), with 81.4% of the very well informed indicating they would like to know more, reducing to 78.4% for those who feel reasonably well informed, 62.3% for those who do not know much about it, and 48.9% for those who have never heard of it.

The most popular source of information on pests and diseases was via traditional media such as TV (68.0%), newspapers (42.8%), radio (21.7%) and magazines or journals (13.2%). A further 19.9% heard about the issue via friends and family, 10.9% from internet searches, 10.7% from staff at visitor centres and 4.5% from work colleagues. Only 3.1% heard of it via Twitter, although 12.8% said they heard of it via other social media. For those who would like to know more about the issue, the most likely sources to be used were internet searches (61%), TV (60%), staff at visitor centres (53.1%) and the FC website (52.6%). They would be least likely to go to Twitter, with just 14.2% saying likely or very likely.

A cumulative odds ordinal logistic regression with proportional odds was run to determine the effect of gender, place identity, membership of environmental groups and woodland visits,^3^ on awareness of tree pests and diseases (Table 1). The assumption of proportional odds was met, as assessed by a full likelihood ratio test comparing the fit of the proportional odds model to a model with varying location parameters, *χ* ^2^(8) = 9.680, *p* = .288. The deviance goodness-of-fit test indicated that the model was a good fit to the observed data, *χ* ^2^(38) = 51.589, *p* = .070, with only 18.3% of cells with zero frequencies. The final model was statistically significant, predicting the dependent variable over and above the intercept-only model, *χ* ^2^(4) = 172.361, *p* < .001. The odds of woodland visitors being aware of tree pests and diseases was 2.744 (95% CI 2.041–3.689) times that for non-visitors, *χ* ^2^(1) = 44.630, *p* < .001.^4^ The odds of environmental group members being aware was 2.352 (95% CI 1.843–3.003) times that of non-members, a statistically significant effect, *χ* ^2^(1) = 47.139, *p* < .001. The odds of respondents with high place identity being aware of tree pests and diseases was 1.588 (95% CI 1.185–2.130) times that of those with low place identity, a statistically significant effect, *χ* ^2^(1) = 9.556, *p* = .002. There were no statistically significant effects of gender on awareness, *χ* ^2^(1) = 2.170, *p* = .141.

**Table 1:** Dependent and explanatory variables used in ordinal logistic regression model to identify predictors of awareness and concern about tree pests and pathogens

	Categories
Dependent variable ‘awareness’	
* ‘British trees, woodlands and forests are currently threatened by a range of newly introduced pests and diseases. Which of the following statements best describes your current level of awareness?’*	1. I have never heard of this problem; 2. I have heard of this problem but do not know much about it; 3. I have heard of this problem and feel I am reasonably well informed; 4. I have heard of this problem and feel I am very well informed.
Dependent variable ‘concern’	
* ‘How concerned are you about the threat to UK trees, woodlands and forests from pests and diseases?’*	1. Not at all concerned; 2. Slightly concerned; 3. Concerned; 4. Very concerned; 5. Extremely concerned
Explanatory variables	
* Gender*	Male or female
* Identity*	High/low place identity
* Environmental group*	Member of environmental or countryside organisation: yes/no
* Woodland visits (on ‘awareness’ model only)*	Woodland visitor: yes/no
* Plant purchasing (on ‘concern’ model only)*	Purchased plants in the last 12 months: yes/no

Gender, income and ethnicity were not related to levels of awareness (Table 6 in “Appendix”). However, respondents over 55 were significantly more likely to have heard of the issue and know something about it compared to younger respondents and awareness appeared to increase significantly with level of education. There was some variation in levels of awareness across geographic regions, with respondents in the East and South East of England significantly more likely to indicate that they feel reasonably well informed. Respondents in London, the East Midlands, North East England and North West England were the most likely to indicate they had never heard of the issue. However, there was no significant difference in terms of levels of concern between regions within England, Scotland or Wales.

A significantly higher proportion of those who indicated they are a member of one of the listed environmental or countryside organisations were aware of the issue. Awareness was also significantly higher amongst respondents who visit woodlands frequently, purchase plants and exchange plants with friends (e.g. gardeners) and collect firewood, have been affected by tree pests and diseases or ‘strongly agreed’ with statements relating to the importance of woodland. While awareness appears to be significantly higher for those that expressed high place identity, levels of place dependence^5^ did not relate to awareness.

### Concern about tree health issues

**Fig. 2:**
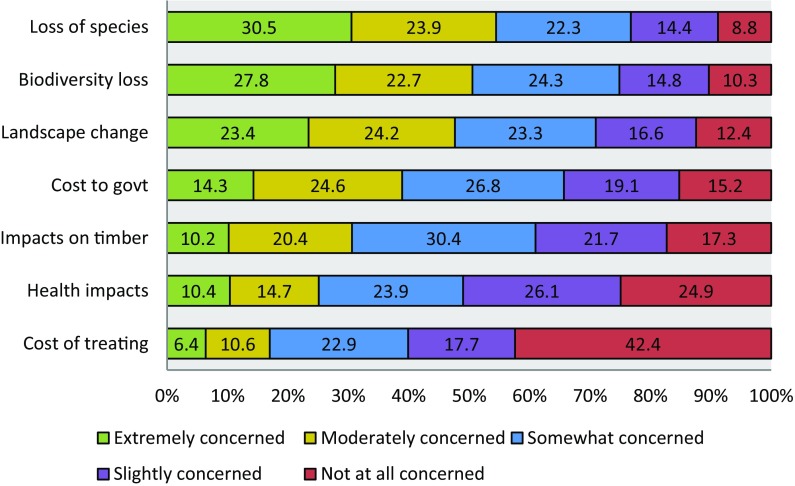
Stated concerns about impacts of tree pests and diseases

Around one in three respondents indicated they were either extremely concerned or very concerned about tree health issues, while only 7.7% were not at all concerned. The highest concern related to the potential loss of a tree species in the UK, with 91.2% indicating that they were either extremely, moderately, somewhat or slightly concerned (Fig. 2). There were also concerns about the impacts on woodland biodiversity (89.7%), change in the landscape where they live (87.6%), the costs to government causing pressure on funding other activities (84.8%), the impacts on commercial timber production (82.7%), the health impacts on themselves and their family (75.1%) and the costs to themselves of treating a diseased tree (57.6%).

Factor analysis revealed that risk concerns fall into two categories (Table 2): Factor 1: concern about the broad threats to public goods and ecosystem services (e.g. loss of a tree species, biodiversity, landscape and the forest as an economic resource); and Factor 2: concern about personal impacts such as the cost of removing or treating an infected tree on their land or the health impacts to themselves or their family. Factor 1 demonstrated the highest percentage of cumulative variance 60.6%, compared to 16.0% for Factor 2.

**Table 2:** Rotated factor loadings for public risk perceptions towards tree pests and diseases

Factors/items	Factor 1	Factor 2
Factor 1: threats to public goods and ecosystem services		
Concern about impacts on woodland biodiversity	.925	.112
Concern about loss of tree species	.891	.152
Concern about landscape change	.855	.230
Concern about impacts on timber production	.707	.432
Concern about costs to government	.682	.418
Factor 2: threat of personal economic and health impacts		
Concern about cost dealing with infected trees	.149	.884
Concern about health impacts on family	.253	.839
Eigenvalue	4.244	1.122
% of cumulative variance	60.6	16.0
Cronbach’s alpha^a^	.910	.756

Factor loadings derived from rotated component matrix using principal component analysis and varimax rotation with Kaiser normalisation

^a^Cronbach’s alpha measures how closely related a set of items are, with coefficients over .7 indicating good internal consistency

A cumulative odds ordinal logistic regression with proportional odds was run to determine the effect of gender, place identity, membership of environmental groups and plant buying, on concern about tree pests and diseases (Table 1). While the full likelihood ratio test comparing the fit of the proportional odds model to a model with varying location parameters suggested the assumption of proportional odds was not met, *χ* ^2^(12) = 26.467, *p* ≤ .009, separate binomial logistic regressions indicated similar odds ratios for each dichotomized cumulative category, thus the assumption of proportional odds was considered met.^6^ The deviance goodness-of-fit test indicated that the model was a good fit to the observed data, *χ* ^2^(56) = 68.679, *p* = .119, with only 8.8% of cells with zero frequencies. The final model statistically significantly predicted the dependent variable over and above the intercept-only model, *χ* ^2^(4) = 108.833, *p* < .001. The odds of environmental group members being concerned about tree pests and diseases was 2.339 (95% CI 1.831–2.989) times that of non-members, a statistically significant effect, *χ* ^2^(1) = 46.170, *p* < .001. The odds of plant buyers being concerned about tree pests and diseases was 1.842, (95% CI 1.505–2.254) times that for those who did not buy plants, *χ* ^2^(1) = 35.224, *p* < .001. The odds of respondents with high place identity being concerned about tree pests and diseases was 1.648 (95% CI 1.230–2.209) times that of those with low place identity, *χ* ^2^(1) = 11.188, *p* = .001. There was no statistically significant effects of gender on concern about tree pests and diseases, *χ* ^2^(1) = .222, *p* = .687.

Cross-tabulations revealed that income, education level, ethnicity and geographic location did not relate to levels of concern (Table 6 in “Appendix”). There was a significant difference between age categories and levels of concern, with older respondents (65+) more likely to be extremely or very concerned than younger respondents. Younger respondents (18–44 years) were more likely than older respondents to be not at all concerned or slightly concerned. Those employed in senior managerial or professional roles and semi-skilled or unskilled manual workers were the least likely to be concerned. Members of environmental or countryside-related organisations were also significantly more likely to express higher levels of concern than non-members. Concern was significantly higher amongst respondents who visit woodlands frequently, purchase plants and exchange plants with friends (e.g. gardeners), collect firewood or ‘strongly agreed’ with statements relating to the importance of woodland. While concern appears to be higher for those that expressed high place identity, levels of place dependence did not influence concern. Those who were more concerned appear to have higher levels of awareness, with 65.1% of those saying they are extremely concerned indicating that they are very well informed about the problem, *χ* ^2^(12) = 491.939, *p* < .001. Only 3.9% of those who have never heard of the problem indicated that they are extremely concerned.

### Willingness to adopt biosecure behaviours

If respondents thought there was a diseased tree on or near their property, most (59.7%) said they would be likely or highly likely to report it to their local authority; 58.1% would talk to family and friends; 49.9% would try to find out more and 43.4% would report it to the FC. Only 20.7% would try to tackle the problem themselves and 16.8% said they would do nothing. Respondents were unlikely to share information about pests and diseases themselves, except for talking with friends and family.

From the survey results, there were indications that there is some public willingness to adopt measures to reduce the spread of pests and diseases. Of the biosecurity actions listed, 66.2% of respondents indicated that they are very likely or likely to avoid bringing plants and wood products into the UK from abroad. A majority (62.6%) said they would buy from trusted local sources, 55.7% would buy plants that are certified as grown in UK, 53.6% would avoid removing soil or leaf litter, 48.5% would clean footwear/bike tyres and 40.7% would take part in surveys to detect early signs. Paying more for plants from accredited sources was least selected/accepted, with just 37.6% saying they would be likely or very likely to do this.

Table 3 indicates the significance of demographic and lifestyle factors on willingess to adopt biosecure behaviours. In summary, female respondents were significantly more likely than males to indicate willingness to adopt biosecure behaviours, as were older respondents between 55 and 75 years old. Place identity and dependence also appear to increase willingness to adopt biosecure behaviours, and respondents with high positive attitudes towards trees and woodlands, those who buy plants, regular woodland visitors and members of environmental organisations were most likely to be willing to adopt biosecure behaviours. Willingness to change plant-buying behaviour reduced with level of education, with those educated to at least degree-level least willing.

**Table 3:** Significance of demographic and lifestyle variables for respondent responses to statements about willingness to adopt biosecure behaviours

Variable	Significance of χ^2^						
	Not import	Buy local	Buy UK	Avoid moving soil	Clean footwear	Surveys	Pay more
Q: How likely are you to do any of the following over the next 12 months?^#^							
Gender	**	***	***	**	*	*	***
Age	***	***	***	***	NS	*	***
Education	NS	***	*	**	NS	NS	*
Income	NS	*	NS	NS	NS	NS	*
Visit	***	***	***	***	***	***	***
Purchased plants from garden centre	***	***	***	***	**	**	***
Identity	***	***	***	*	NS	*	***
Member	***	***	***	***	NS	***	***
Region	*	NS	NS	NS	NS	NS	NS
Dependence	**	**	*	NS	*	***	***
Importance of woodland	***	***	***	***	***	***	***

*** *p* < .001; ** *p* < .005; * *p* < .05; *NS* not significant

^#^Response options: (1) not at all concerned; (2) slightly concerned; (3) concerned; (4) very concerned; (5) extremely concerned

### Changes in public attitudes and awareness: 2013–2016

There was some difference in attitudes towards trees and woodlands between the two datasets. Respondents in the 2013 sample were more likely than 2016 respondents to ‘strongly agree’ that woodlands provide economic income and jobs (*χ* ^2^(4) = 95.524, *p* < .001), are important places for wildlife (*χ* ^2^(4) = 37.372, *p* < .001) and places where the community can gather (*χ* ^2^(4) = 24.792, p < .001).

There has been a statistically significant decline in awareness of tree pests and diseases since 2013 (*χ* ^2^(4) = 35.822, *p* < .001). While 16.4% of respondents in 2013 said they had never heard of the problem, this figure had risen to 21.0% by 2016. Those who believed they were reasonably well informed declined from 27.6% in 2013 to 18.7% in 2016.

Despite the overall decline in stated awareness, there was some variability in awareness of a range of pests and diseases. In 2016 there was less awareness of Oak processionary moth, *Phytophthora ramorum* and Ash dieback than in 2013. However, there was greater awareness of Emerald ash borer, Dothistroma needle blight, Great spruce bark beetle, Chestnut blight and Asian longhorn beetle. Awareness of Dutch elm disease and Acute oak decline appeared to be consistent across both datasets.

Awareness of control measures also declined between 2013 and 2016. Those who had not heard about controls on imports of trees and plants increased from 31.3% in 2013 to 68.9% in 2016. There was a similar decline in awareness about restrictions on the movement of infected or infested wood or timber (43.9% in 2013 had not heard of it, 75.4% in 2016); the use of chemical treatments (36.6% in 2013, 69.6% in 2016) and new research to find out more (44.1% in 2013, 70.2% in 2016).

In both years, the majority of respondents suggested the FC has primary responsibility for managing and controlling tree pests and diseases (2013: 33.1%; 2016: 35.4%). However, in 2013 a further 32.8% indicated Defra has primary responsibility (this option was not provided in the 2016 survey). The ‘don’t know’ respondents increased from 16.6% in 2013 to 21.4% in 2016. If respondents thought there was a diseased tree on or near their property the most likely course of action in both 2013 and 2016 was to contact the local authority.

The importance of the media as a source of knowledge was apparent in both 2016 and 2013, with 57.7% indicating they had heard about the issue via radio or TV and 16.2% via newspapers in 2013 (in 2016 the figures were 68.0% for TV, 21.7% for newspapers and 42.8% for radio). In 2013, 77.2% of respondents were interested in knowing more, compared to 63.1% in 2016 (*χ* ^2^(4) = 52.913, *p* < .001). There were similarities in the sources identified to provide further information: internet searches, print and broadcast media, staff at visitor centres and the FC website. Only 11.4% of 2013 respondents said they would use social media to find out more (compared to 14.2% in 2016 who indicated they would use Twitter to find out more).

**Fig. 3:**
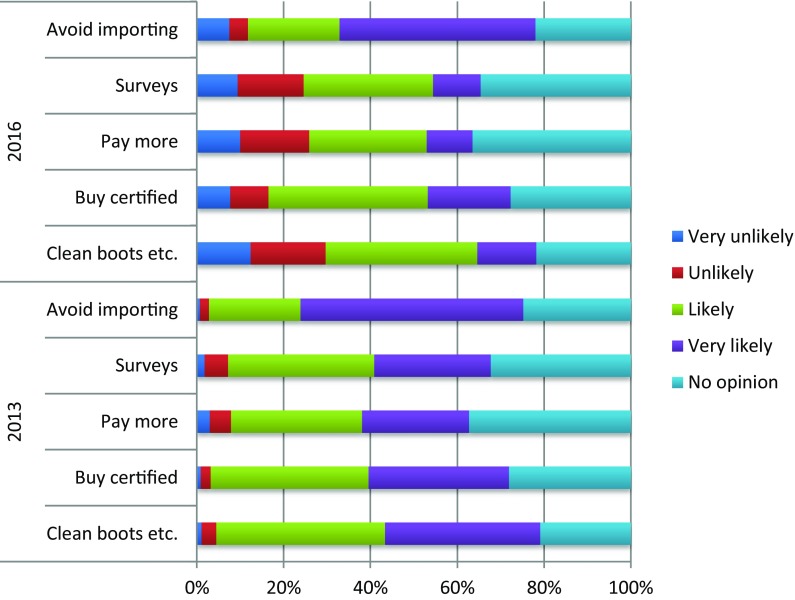
Percentage of respondents in 2013 and 2016 who are willing to adopt biosecure behaviours

Levels of concern also appear to have declined between 2013 and 2016. In 2013, 78.1% of respondents indicated they were either ‘concerned’ or ‘very concerned’. In 2016 58.8% of respondents indicated they were ‘concerned’, ‘very concerned’ or ‘extremely concerned’. Further, respondents in 2013 were significantly more willing to adopt biosecure behaviours than those in 2016, such as cleaning footwear, tyres or dogs’ paws (*χ* ^2^(4) = 316.923, *p* < .001), buying UK certified plants (*χ* ^2^(4) = 136.658, *p* < .001), paying more for plants from an accredited source (*χ* ^2^(4) = 172.256, *p* < .001), taking part in pest/disease detection surveys (*χ* ^2^(4) = 188.132, *p* < .001) and avoiding bringing plants back from trips abroad (*χ* ^2^(4) = 72.668, *p* < .001) (Fig. 3).

## Discussion

### Public awareness and concern about tree pests and diseases

In line with other recent surveys (e.g. Fuller et al. 2016; Marzano et al. 2016; McFarlane et al. 2006), our findings indicate generally low levels of awareness and knowledge of tree pests and diseases, but higher levels of stated concern. This may reflect a tendency for people to be more concerned about unfamiliar risks or those they know little about, risks that may have effects that are delayed in time and where there is scientific uncertainty (Renn 2008; Slovic et al. 1980; Williamson and Weyman 2005). In our study, of particular note is the nature of ‘concern’, which related to public good impacts rather than personal impacts. For example, respondents were more concerned about threats to biodiversity, recreational opportunities, landscape and the loss of a tree species, rather than the potential economic or health impacts on themselves which is prominent in studies of technological risks (Bickerstaff et al. 2008; Lima and Marques 2005). A note of caution is needed in the interpretation here. While our study, along with previous surveys cited, demonstrated low awareness but higher concern, this may simply be a function of the nature of the question. ‘Concern’ questions are by their nature much more subjective (i.e. ‘how concerned are you?’), in contrast to the more objective ‘awareness’ questions (i.e. ‘have you heard of?).

Membership of environmental organisations and high place identity appeared to be more important predictors of concern and awareness than socio-demographic factors. Intuitively one would expect those interested in the environment to be more engaged with tree health issues, but the finding that there is a correlation between place identity and tree health perceptions is of note. This finding aligns with Lima and Marques’ (2005) study about the siting of a new waste incinerator, where they found that both proximity to the hazard and place identity are likely to amplify risk concerns. Conversely, some scholars suggest that high attachments to place may in fact attenuate risk concerns over nearby hazards as individuals seek to avoid acknowledgement of a potential risk associated with a valued place and over time accept the risk as part of the identity of the place (Bickerstaff et al. 2008; Burningham and Thrush 2004; Venables et al. 2012). Indeed, our finding that respondents in 2016 were less concerned than those in 2013 may reflect a growing acceptance of tree health risks from a peak in public attention in the wake of the high profile Ash dieback outbreak in late 2012. Over time people adapt to the presence of a hazard and normalise the risk (Barnett and Breakwell 2003; Lima and Marques 2005), with their attention moving on to other novel risks appearing on the horizon. Our findings are supported by results in the ‘Public Opinion of Forestry’ survey carried out by the FC which found that the percentage of respondents who expressed concern or were willing to look out for and report sightings of pests and diseases had declined between 2013 (FC 2013) and 2015 (FC 2015). Clearly, further research that considers place-based dimensions in the experience and perception of tree pests and diseases is warranted (see, for example, Palmer et al. (2014) application of a ‘relational place-making’ framework to explore adaptive capacity in the context of the Asian longhorn beetle outbreak in Worcester, Massachusetts, USA).

The most widely recognised tree pest or disease was Dutch elm disease, a fungal pathogen that caused widespread losses of elms in the 1970s. Around 95% of respondents over the age of 55 who had heard of tree pests and diseases were aware of Dutch elm disease, compared to just 40% of 18–24 year olds. However, the same phenomenon is demonstrated for the more recent outbreak of Ash dieback with older respondents being more aware (73% of over 75s, compared to 20% of 18–24 year olds), suggesting that age is a more important predictor of awareness than ‘living through’ an outbreak. Older respondents in our study also expressed higher concern about tree pests and diseases than younger respondents.

### Risk communication, responsibility and public engagement with tree health issues

Our study suggests individuals with higher levels of knowledge about invasive tree pests and diseases are more likely to be attentive to tree health issues and adopt biosecure behaviours. Given that awareness is generally low, and appears to have declined since the peak of the public attention on the Ash dieback outbreak in 2012, there is a need for continued public engagement and risk communication. This would better equip the general public with the necessary information to detect and respond to occurrences of pests and diseases. Awareness levels in 2013 are likely to have been influenced by the high media profile of Ash dieback in late 2012, with almost 75% of those respondents indicating they first heard of the issue of tree pests and diseases via the radio, television and newspapers. Since its peak in late 2012, media attention to Ash dieback has declined (Fellenor et al. 2017), with sporadic coverage alongside other tree pest and disease outbreaks. The role of the media in the social construction of risks, both in terms of how journalists frame events and as a primary tool for how the public learns about risk events, is well documented (Flynn et al. 1998; Höijer 2010; Hornig 1993; Lewis and Tyshenko 2009) and our study concurs that traditional media is an important source of information for finding out about tree pests and diseases.

Compared to expressed levels of concern, willingness to adopt biosecure behaviours is fairly low, with less than half indicating they would clean their footwear in order to reduce the likelihood of spreading tree pathogens. More promising is a willingness to change plant-buying behaviours. While few (38%) indicated they would be willing to pay more for plants from an accredited source, almost two-thirds would buy from a local trusted source and refrain from bringing back plants from abroad. Women, older people, those engaged in environmental activities and those with high attachments to place were identified as the most willing.

Although respondents such as woodland visitors and gardeners demonstrated higher levels of awareness and concern, together with a willingness to adopt biosecure behaviours, they are also the most likely to be engaged in activities which can potentially lead to the spread of pests and diseases, such as through the purchase of plants or inadvertent movement of organisms from one woodland to another on footwear, bike tyres or dogs’ paws. Therefore, in the short-term it may be more effective to target these groups to raise awareness about the importance of adopting appropriate biosecurity measures, such as through the FC’s recent “Keep it Clean”^7^ campaign.

The importance of membership of environmental and countryside organisations in shaping views suggests these social groups are likely to be important linchpins in risk communication about tree health risks. Organisations such as the Royal Horticultural Society, the Ramblers Association and the horticultural industry itself, may be well-placed to provide enhanced information to members and others about responsible plant purchasing, practical measures to avoid spreading disease, how to detect disease and where to report it. These organisations may be more trusted, understand their members better and have a greater access to particular social groupings (Quine et al. 2011). Further, the mixed views about who has primary responsibility for dealing with tree pests and diseases and who to contact if a pest or disease is detected suggests that the general public may need clearer and more specific guidance on how to respond.

In their study of the threats facing UK biodiversity, Sutherland et al. (2008) identified the decline in people’s engagement with nature as having the potential to reduce environmental knowledge and concern. If, as our study suggests, awareness of tree health issues is enhanced through engagement with nature then the apparent decline in engagement is of concern. This may reduce awareness and could impact on public support for the prevention and management of tree pests and diseases in the future (Bayliss and Potter 2013). It may further reduce the willingness of publics to recognise their role in responding to pest and disease outbreaks and adopting biosecure behaviours themselves.

## Conclusion

The results of our survey found that one in three respondents were either extremely concerned or very concerned about the health of UK trees, forests and woodlands, and less than a tenth were not at all concerned. However, there was low awareness and knowledge about tree pests and diseases, with 21% of respondents indicating that they had never heard of the issue. A key finding of this study is that attentiveness to tree health issues declined between 2013 and 2016. However, while no pre-Ash dieback baseline for public perceptions exists, it is likely that the 2013 data reflect heightened public attention to the Ash dieback outbreak at that time.

Further work is needed to explore and better understand the temporal and spatial nature of public concern around tree health, especially how outbreaks are experienced, perceived and produce local responses (such as Porth et al.'s (2015) focus on local residents’ experience of the Asian longhorn beetle outbreak in Kent, UK). As Irwin (2001) points out: “Environmental problems do not sit apart from everyday life (as if they were discrete from other issues and concerns) but instead are accommodated within (and help shape) the social construction of local reality” (p. 175). There is considerable scope for applying place-based approaches for understanding the particular socio-cultural and spatial contexts within which risk perceptions are constructed (Henwood et al. 2008; Parkhill et al. 2010).

The decline in attentiveness suggests further efforts are required to raise the interest in tree health issues outside the ‘peaks’ of public attention, through enhanced risk communication. While it may be appropriate to target ‘higher risk’ and ‘more willing’ groups, such as those engaged in environmental activities, members of environmental groups or gardeners, in the short-term, we suggest there is a need to encourage broader public dialogue around the issue of plant biosecurity and the practices of the horticultural and tree nursery industry, alongside efforts to influence public behaviour. If the public are attentive to the pathways and drivers for invasive pest and disease introduction, and if the government is sensitive to public concern, then a more attentive public is likely to result in not only individual behaviour change but pressure to enhance the regulation and behaviour of the plant trade industry.

## Appendix: Supplementary material

See Tables 4, 5 and 6.

**Table 4:** Sample profile in terms of gender, age, employment status, income and education level of survey respondents

Variable/category	Sample (n = 1334) %
Gender	
Male	48.1
Female	51.9
Age group	
18–24	11.5
25–34	16.8
35–44	16.4
45–54	18.2
55–64	14.2
65+	22.9
Employment status	
Retired	25.6
Junior managerial administrative or professional roles	19.9
Intermediate managerial, administrative or professional roles	10.6
Semi-skilled or unskilled manual workers	9.3
Skilled manual workers	6.5
Homemakers	6.5
Students	5.2
Permanently unemployed (e.g. sick, independent means)	4.8
Senior managerial, administrative, professional or business owners	3.5
Carers	2.2
Other	1.6
Income	
<£5200	10.2
£5200–£10,399	11.8
£10,400–£15,599	15.4
£15,600–£20,799	16.8
£20,800–25,999	13.7
£26,000–£31,199	9.2
£31,200–£36,399	6.2
£36,400–£51,999	9.7
>£52,000	6.2
Education level	
School level qualifications (e.g. GCSEs, O-levels)	24.6
Post-secondary level qualification (e.g. A-levels)	22.6
University level qualification (e.g. degree)	22.5
Vocational qualifications	10.9
Higher degrees (e.g. Masters, PhD)	8.2
Professional qualifications	6.6
Other (e.g. no qualifications)	2.5
Apprenticeships	2.2

**Table 5:** Rotated factor loadings for place attachment dimensions, including mean and standard deviation (*n* = 1334)

Factors/items	Factor 1	Factor 2	Median^1^	SD
Factor 1: place identity			3.67	.92
‘This area is very special to me’	.948	−.031	4.00	1.03
‘I identify strongly with this area’	.936	−.019	4.00	1.03
‘I am very attached to this area’	.880	.056	4.00	1.04
‘This area means a lot to me’	.857	.093	4.00	1.02
‘I feel this area is a part of me’	.946	−.081	4.00	.98
‘Living in this area says a lot about who I am’	.542	.342	3.00	1.05
Factor 2: place dependence			3.00	.96
‘I would not substitute any other area for doing the types of thing that I do here’	−.073	.958	3.00	1.08
‘Doing the activities I enjoy in this area is more important to me than doing them in any other place’	−.020	.917	3.00	1.07
‘No other area can compare to this area’	−.037	.911	3.00	1.12
‘I get more satisfaction out of living in this area than any other place’	.126	.805	3.00	1.11
‘This area is the best place for doing the things I like to do’	.291	.604	4.00	1.07
Eigenvalue^2^	7.538	1.242		
% of cumulative variance	68.5	11.3		

KMO = .951; Bartlett’s test of sphericity *χ* ^2^(55) = 14,494.766, *p* < .001

Factor loadings derived from rotated pattern matrix using principal component analysis and oblimin rotation with Kaiser normalisation (rotation involves rotation of the axes in a factor analysis so that clusters of items fall as close to them as possible in order to aid interpretation). The final anti-image matrix showed no large values, the Bartlett test of sphericity Chi square value of 14,494.766 was significant (<.001), the overall measure of sampling adequacy was .951 and the communality for each variable was greater than .50, thus confirming that the data was adequate for factor analysis

^1^Mean scores range from 1 to 5 and reflect the summed scales of the Likert scale response categories of 1—strongly disagree, 2—disagree, 3—no opinion, 4—agree, 5—strongly agree

^2^Eigenvalues reflect the amount of variation in the data accounted by each factor, with eigenvalues over 1 typically determining the number of factors to be selected (Tabachnick and Fidell 1996)

**Table 6:** Chi square tests and significance of demographic and lifestyle variables for respondent responses to statements about level of awareness of and concern about tree pests and pathogens

Variable	Awareness^1^ *χ* ^2^	Concern^2^ *χ* ^2^
Gender	4.298	4.054
Age	127.544***	64.380***
Education	34.748*	54.487
Job	91.765***	100.247***
Income	31.396	43.438
Visit	138.082***	150.829***
Activity		
Visited wood to walk dog	29.567***	28.102***
Visited wood for recreation	16.772**	25.638***
Visited garden or park	15.866**	15.621**
Collected firewood	29.567***	9.773*
Purchased plants from garden centre	64.562***	27.423***
Exchange plants with friends	36.603***	14.109**
Identity	9.249*	20.770***
Member	52.477***	67.974***
Ethnicity	30.029	38.103
Region	51.804*	51.068
Dependence	7.664	6.898
Importance of woodland		
For economic income & jobs	131.462***	199.090***
For wildlife	45.234***	117.866***
For people to enjoy themselves	56.738***	170.355***
For keeping fit and exercise	36.223***	122.698***
Make area nice to live	56.660***	152.474***
For learning about environment	65.274***	197.153***
For learning about local culture	71.765***	179.573***
For community to come together	84.926***	185.845***

*** *p* < .001; ** *p* < .005; * *p* < .05

^1^Q: British trees, woodlands and forests are currently threatened by a range of newly introduced pests and diseases. Which of the following statements best describes your current level of awareness?

^2^Q: How concerned are you about the threat to UK trees, woodlands and forests from pests and diseases?

## Footnotes

1 Biosecure behaviours in this context may include cleaning footwear, dogs’ paws and bicycles after woodland visits, avoiding bringing plant material into the UK from trips abroad, ensuring that plant purchases come from certified disease/pest-free sources, avoiding moving soil or leaf litter between sites, and so on.

2 The eleven place attachment Likert items used (see Table 5 in “Appendix”) were a sub-set of statements developed and validated in previous studies (e.g. Raymond et al. 2010; Williams et al. 1992; Williams and Vaske 2003). These statements were subjected to principal component analysis in SPSS, confirming two factors representing (a) place identity and (b) place dependence, with a cumulative variance of 68.5 and 11.3% respectively. The statements all have very high loadings and each statement is related to either place identity or place dependence (see Table 5 for further details and explanation of the factor analysis).

3 Preliminary models including the explanatory variables *age, education, location* and *plant purchasing* were tested, but these variables were excluded in the final model due to a high number of cells with zero frequencies and the assumption of proportional odds not being met.

4 Although the results for the woodland visit variable were significant, a binomial logistic regression suggested that the assumption of similar odds for this variable might not be tenable, with coefficients of 2.786, 2.813 and 1.114 on the cumulative splits of the ordinal dependent variable ‘awareness’.

5 See Table 1 for survey statements relating to place identity and place dependence.

6 Proportional odds states that the estimated parameters (B) are equal for each binomial logistic regression on each dichotomized cumulative category, thus the odds ratio (Exp(B)) should also be similar.

7
http://scotland.forestry.gov.uk/keep-it-clean.

## References

[CR2] Barnett J, Breakwell G (2003). The social amplification of risk and the hazard sequence: the October 1995 oral contraceptive pill scare. Health Risk Soc.

[CR3] Bayliss H, Potter C (2013) Survey of public awareness and understanding of introduced tree pests and diseases in the United Kingdom, Draft Working Paper 4. Imperial College London, Defra Project TH0104

[CR4] Bickerstaff K, Lorenzoni I, Pidgeon NF, Poortinga W, Simmons P (2008). Reframing nuclear power in the UK energy debate: nuclear power, climate change mitigation, and radioactive waste. Public Underst Sci.

[CR5] Boyd IL, Freer-Smith PH, Gilligan CA, Godfray HCJ (2013). The consequence of tree pests and diseases for ecosystem services. Science.

[CR6] Burningham K, Thrush D (2004). Pollution concerns in context: a comparison of local perceptions of the risks associated with living close to a road and a chemical factory. J Risk Res.

[CR7] FC (2013). Public opinion of forestry 2013, UK and England.

[CR8] FC (2015). Public Opinion of Forestry 2015, UK and England.

[CR9] Fellenor J, Barnett J, Potter C, Urquhart J, Mumford J, Quine CP (2017). The social amplification of risk on Twitter: the case of ash dieback disease. J Risk Res.

[CR10] Flint CG (2007). Community perspectives on spruce beetle impacts on the Kenai Peninsula, Alaska. For Ecol Manag.

[CR11] Flynn J, Peters E, Mertz CK, Slovic P (1998). Risk, media and stigma at Rocky Flats. Risk Anal.

[CR12] Freer-Smith PH, Webber JF (2015) Tree pests and diseases: the threat to biodiversity and the delivery of ecosystem services. Biodivers Conserv. doi:10.1007/s10531-015-1019-0

[CR13] Fuller L, Marzano M, Peace A, Quine CP, Dandy N (2016). Public acceptance of tree health management: results of a national survey in the UK. Environ Sci Policy.

[CR14] Haack RA, Law KR, Mastro VC, Ossenbruggen HS, Raimo BJ (1997). New York’s battle with the Asian long-horned beetle. J For.

[CR15] Henwood KL, Pidgeon NF, Sarre S, Simmons P, Smith N (2008). Risk, framing and everyday life: methodological and ethical reflections from three sociocultural projects. Health Risk Soc.

[CR16] Heuch J (2014). What lessons need to be learnt from the outbreak of ash dieback disease, Chalara fraxinea in the United Kingdom?. Arboric J Int J Urban For.

[CR17] Höijer B (2010). Emotional anchoring and objectification in the media reporting on climate change. Public Underst Sci.

[CR18] Hornig S (1993). Reading risk: public response to print media accounts of technological risk. Public Underst Sci.

[CR19] Irwin A (2001). Sociology and the environment: a critical introduction to society, nature and knowledge.

[CR20] Jorgensen B, Stedman RC (2001). Sense of place as an attitude: lakeshore owners’ attitudes toward their properties. J Environ Psychol.

[CR22] Lewis RE, Tyshenko MG (2009). The impact of social amplification and attenuation of risk and the public reaction to mad cow disease in Canada. Risk Anal.

[CR23] Liebhold AM, Brockerhoff EG, Garret LJ, Parke JL, Britton KO (2012). Live plant imports: the major pathway for forest insect and pathogen invasions of the US. Front Ecol Environ.

[CR24] Lima ML, Marques S (2005). Towards successful social impact assessment follow-up: a case study of psychosocial monitoring of a solid waste incinerator in the North of Portugal. Impact Assess Proj Apprais.

[CR25] Loewenstein G, Mather J (1990). Dynamic processes in risk perception. J Risk Uncertain.

[CR26] Marzano M, Dandy N, Bayliss HR, Porth E, Potter C (2015). Part of the solution? Stakeholder awareness, information and engagement in tree health issues. Biol Invasions.

[CR27] Marzano M, Dandy N, Papazova-Anakieva I, Avtzis D, Connolly T, Eschen R, Glavendekic M, Hurley B, Lindelow A, Matosevic D, Tomov R, Vettraino AM (2016). Assessing awareness of tree pests and pathogens amongst tree professionals: a pan-European perspective. For Policy Econ.

[CR28] Masuda JR, Garvin T (2006). Place, culture and the social amplification of risk. Risk Anal.

[CR29] Mccombs M, Reynolds A, Bryant J, Zillmann D (2002). News influence on our pictures of the world. Media effects: advances in theory and research.

[CR30] McFarlane BL, Stumpf-Allen RCG, Watson DO (2006). Public perceptions of natural disturbance in Canada’s national parks: the case of the mountain pine beetle (*Dendroctonus ponderosae* Hopkins). Biol Conserv.

[CR31] Molnar JJ, Schelhas J, Holeski C (2003). Controlling the Southern Pine Beetle: small landowner perceptions and practices, Bulletin 649.

[CR32] Palmer S, Martin D, DeLauer V, Rogan J (2014). Vulnerability and adaptive capacity in response to the Asian longhorned beetle infestation in Worcester, Massachusetts. Hum Ecol.

[CR33] Parkhill KA, Pidgeon N, Henwood KL, Simmons P, Venables D (2010). From the familiar to the extraordinary: local residents’ perceptions of risk when living with nuclear power in the UK. Trans Inst Br Geogr.

[CR34] Porth EF, Dandy N, Marzano M (2015). “My garden is the one with no trees:” residential lived experiences of the 2012 Asian longhorn beetle eradication programme in Kent, England. Hum Ecol.

[CR35] Potter C, Urquhart J (2017). Tree disease and pest epidemics in the Anthropocence: an analysis of drivers, impacts and policy responses in the UK. For Policy Econ.

[CR36] Quine CP, Barnett J, Dobson AM, Marcu A, Marzano M, Moseley D, O’Brien L, Randolph SE, Taylor JL, Uzzell D (2011). Frameworks for risk communication and disease management: the case of Lyme disease and countryside users. Philos T Roy Soc B.

[CR37] Raymond CM, Brown G, Weber D (2010). The measure of place attachment: personal, community and environmental connections. J Environ Psychol.

[CR38] Relph E (1976). Place and placelessness.

[CR39] Renn O (2008). Risk governance: coping with uncertainty in a complex world.

[CR40] Runberg DM (2011) Educating Pacific Northwest campers on the risk of spreading invasive forest pests through firewood: developing a mental model, Dissertation submitted for Master of Public Policy. Oregon State University, Eugene, Oregon, USA

[CR41] Sheremet O, Healey JR, Quine CP, Hanley N (2017). Public preferences and willingness to pay for forest disease control in the UK. J Agric Econ.

[CR42] Slovic P, Fischoff B, Lichtenstein S, Schwing RC, Albers WA (1980). Facts and fears: understanding perceived risk. Societal risk assessment: how safe is safe enough.

[CR43] Sutherland WJ, Bailey MJ, Bainbridge IP (2008). Future novel threats and opportunities facing UK biodiversity identified by horizon scanning. J Appl Ecol.

[CR60] Tabachnick BG, Fidell LS (1996) Using multivariate statistics. Harper Collins, New York

[CR44] Timotijevic L, Barnett J (2006). Managing the possible health risks of mobile telecommunications: public understandings of precautionary action and advice. Health Risk Soc.

[CR45] Tomlinson I, Potter CA (2010). Too little, too late? Science, policy and Dutch Elm Disease in the UK. J Hist Geogr.

[CR46] Tuan Y-F (1974). Space and place: the perspective of experience.

[CR47] Venables D, Pidgeon N, Parkhill KA, Henwood KL, Simmons P (2012). Living with nuclear power: sense of place, proximity, and risk perceptions in local host communities. J Environ Psychol.

[CR48] Washer P (2011). Lay perceptions of emerging infectious diseases: a commentary. Public Underst Sci.

[CR49] Williams DR, Vaske JJ (2003). The measurement of place attachment: validity and generalizability of a psychometric approach. For Sci.

[CR50] Williams DR, Patterson ME, Roggenbuck JW, Watson AE (1992). Beyond the commodity metaphor: examining emotional and symbolic attachment to place. Leis Sci.

[CR51] Williamson J, Weyman A (2005). Review of the public perceptions of risk, and stakeholder engagement.

